# Controlled patient blood management under visceral perfusion in open treatment of complex aortic pathologies

**DOI:** 10.1016/j.xjtc.2025.11.002

**Published:** 2025-11-11

**Authors:** Melanie Rusch, Grischa Hoffmann, Nawar Alasad, Rene Rusch

**Affiliations:** Department of Vascular and Endovascular Surgery, University Hospital of Schleswig-Holstein, Kiel, Germany

**Figure:**
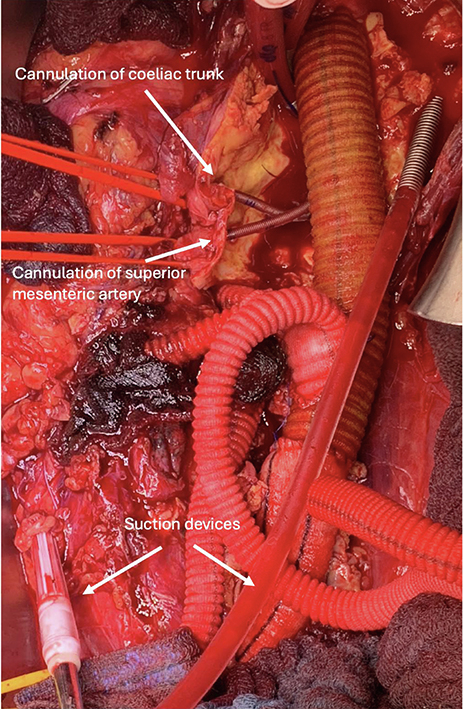
Surgical cannulation technique and various suction devices for direct blood retransfusion.

**Central Message**

The use of an open extracorporeal system in complex aortic surgery stabilizes hemodynamics and minimizes bleeding complications by improved intraoperative blood management.

Complex aortic pathologies, in particular thoracoabdominal aortic aneurysm (TAAA), are life-threatening diseases and one of the most common causes of death from the age of 55 years.^1^ Open surgery was the gold standard in the treatment of TAAA for a long time, but new endovascular alternative procedures have become established in the last 2 decades.^2^ Coselli and colleagues^1^ describe the decision for open or endovascular treatment of TAAA based on the patient's health status and comorbidities, as well as the surgeon's expertise with the respective technology.^1^ Despite various protective measures during surgical treatment, renal and visceral ischemia remain the most common complications, along with high bleeding complications.^1^^,^^2^ Therefore, it is essential to ensure sufficient intraoperative visceral perfusion and controlled hemodynamics, even in cases of higher blood loss. The loss of coagulation components is an especially relevant outcome factor and can lead to increased mortality if blood management is inappropriate.^3^ This technical approach describes the efficacy of an open extracorporeal circulation system in the surgical treatment of TAAA for selective organ perfusion with simultaneous intraoperative blood retransfusion.

## Surgical Technique

**Figure 1:**
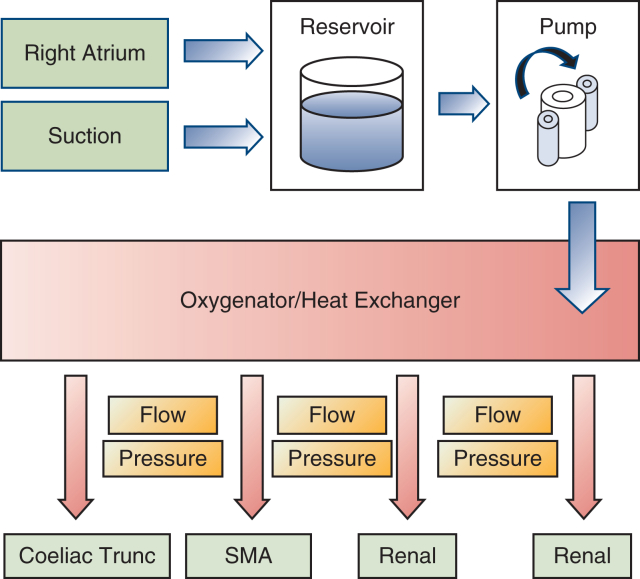
Schematic illustration of the intraoperative setup with the open extracorporeal circulation system. *SMA*, Superior mesenteric artery.

**Figure 2:**
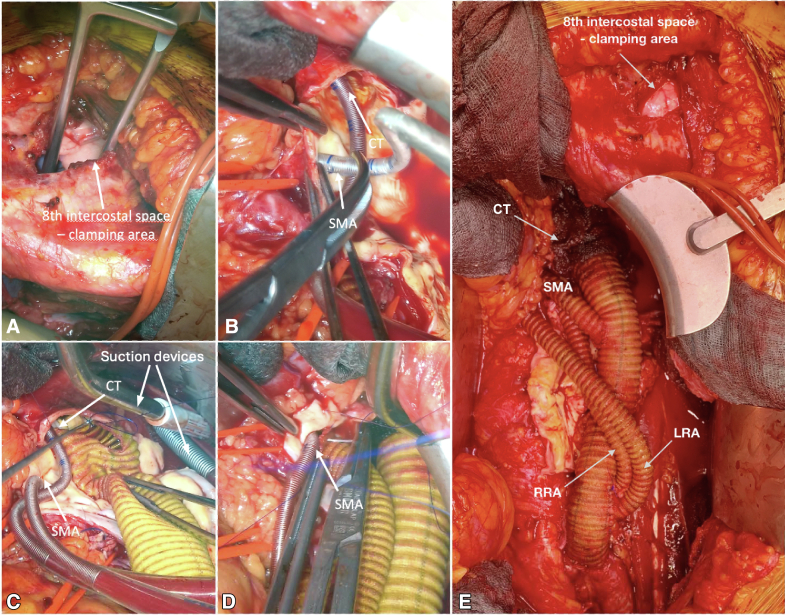
Operative and cannulation technique: (A) crossclamping and (B) selective cannulation of superior mesenteric artery and coeliac trunk. Proximal anastomosis with different suction devices (C) and (D) bypass to the superior mesenteric artery during selective organ perfusion. E, Final result (left and right renal arteries). *CT,* Coeliac trunk; *LRA,* left renal artery; *RRA,* right renal artery; *SMA,* superior mesenteric artery.

Between 2020 and 2022, 7 patients with TAAA Crawford IV were included in this case series. All procedures were performed in accordance with the ethical standards of the institutional or national research committee and approved by the Ethics Committee of the University Hospital of Schleswig-Holstein (D495/19, September 1, 2020). Patient selection was performed according to the current European Society for Vascular Surgery guidelines and after interdisciplinary discussion.^4^ The patients provided informed written consent for the publication of the study data. Replacement of the aorta was performed by implantation of a polyester graft and visceral debranching. The extracorporeal circulation system (Maquet HL20) is primed with Ringer's lactate. Venous outflow drains together with 2 flexible suction devices and 1 rigid suction device are placed into a reservoir followed by 3 roller pumps that pump the blood via a combined heat exchanger and membrane oxygenator (Trilly Infant-Pediatric). Both visceral and renal perfusion are carried out individually via separate roller pumps and Y-bifurcated inflow lines equipped with flow and pressure sensors (ELSA, Transonic Systems) to monitor selective organ perfusion. After systemic heparinization (activated clotting time 350-450 seconds), a 21F venous canula (LivaNova) is advanced percutaneously via the left groin into the right atrium with concomitant arterial cannulation with a 12F canula (DLP, Medtronic). This increased activated clotting time is necessary to avoid clotting of the oxygenator. After supra-coeliac crossclamping and aneurysm opening, two 8F arterial cannulas are inserted into the renal arteries with flow rates of 250 to 300 mL (DLP, Medtronic), secured by encircling vessel loops. After insertion of 2 ballon-tipped, flexible 12F cannulas (TrueFlow RDB, Med Europe) into the coeliac trunk and the superior mesenteric artery, visceral perfusion is initiated with flow rates of 600 to 850 mL each. Pump flows are maintained between 1.5 and 2.0 L/min with a pressure of 60 to 80 mm Hg over the perfusion lines with a mild hypothermia of 35°C (Figure 1). Renal perfusion is monitored by near-infrared spectroscopy monitoring. Relative changes of near-infrared spectroscopy monitoring values can be addressed by change of flow rates. A multibranched graft is implanted with bypasses to the coeliac trunk, superior mesenteric artery, and renal arteries (Figure 2). By sequential declamping and keeping the perfusion catheters in place while constructing the anastomoses, the organ ischemia is kept to a minimum. While the selective inflow catheters are removed after creation of the corresponding bypass anastomosis, the arterial and venous groin canulation remain until the multibranched graft is completely declamped. This allowed significant blood loss to be absorbed and immediately retransfused directly through the venous cannula as inflow.

The use of the open extracorporeal circulation system was safely feasible without any intraoperative complications in all patients undergoing aortic surgery. Selective step-by-step crossclamping times ranged between 30 and 60 minutes. In the postoperative course, only mild lactate increases were observed, which were normal at the time of discharge. There were no postoperative bleeding complications in any of the patients. No deaths occurred during the 30-day interval; 2 patients with preoperative renal impairment required temporary dialysis, which was no longer necessary at discharge.

## Discussion

Compared with traditional closed-loop techniques such as partial left-sided heart bypass, an open extracorporeal circulation system offers continuous visceral perfusion and immediate retransfusion of intraoperative blood loss, resulting in improved blood management with preservation of blood components such as coagulation factors and reduced coagulation products during the postoperative period. All patients routinely underwent at the end of the operation and during intensive care rotational thromboelastometry to assess their coagulation status. With adapted coagulation management, normalized values were consistently observed, and during the postoperative period, no patients reported significant bleeding or hemodynamic incidents. Reduced blood transfusions, avoidance of postoperative bleeding, and optimized volume therapy appear to offer a slight advantage in terms of mortality and complication rates, which is also supported by the literature.^5^ Shorter operating times with reduced ischemia periods and lower transfusion requirements can improve outcomes. The European Society for Vascular Surgery guidelines support the centralization of complex aortic pathologies in specialized high-volume centers, describing that the experience of the center and the respective level of surgical training play an essential role.^4^ Hospitals with a low number of cases are unable to fulfill these conditions; therefore, these open approaches are reserved for high-level facilities.

## Conclusions

The use of an open extracorporeal system not only supports continuous renal and visceral perfusion but also demonstrates high efficacy in optimizing intraoperative blood management. Effective intraoperative retransfusion of significant blood loss and associated coagulation factors resulted in stabilized hemodynamics and reduced bleeding complications. Nevertheless, the use of these systems requires advanced experience and should be performed at specialized aortic centers. Further randomized studies on the optimization of blood management are essential for future application.

## Conflict of Interest Statement

The authors reported no conflicts of interest.

The *Journal* policy requires editors and reviewers to disclose conflicts of interest and to decline handling or reviewing manuscripts for which they may have a conflict of interest. The editors and reviewers of this article have no conflicts of interest.
